# Identification of proteins involved in the pancreatic exocrine by exogenous ghrelin administration in Sprague-Dawley rats

**DOI:** 10.1186/2055-0391-56-6

**Published:** 2014-06-05

**Authors:** Kyung-Hoon Lee, Tao Wang, Yong-Cheng Jin, Sang-Bum Lee, Jin-Ju Oh, Jin-Hee Hwang, Ji-Na Lim, Jae-Sung Lee, Hong-Gu Lee

**Affiliations:** Department of Animal Science and Technology, College of Animal Bioscience & Technology, Konkuk University, 120 Neungdong-ro, Seoul, Gwangjin-gu, 143-701 South Korea; Department of Animal Science, College of Animal Science and Veterinary Medicine, Jilin University, Changchun, 130062 P. R. China

**Keywords:** Alfa-amylase activity, Cholecystokinin, Ghrelin, Pancreatic exocrine, Sprague-Dawley rats, Two-dimensional gel electrophoresis

## Abstract

The aims of study were to investigate the effects of intraperitoneal (i.p.) infusion of ghrelin on pancreatic α-amylase outputs and the responses of pancreatic proteins to ghrelin that may relate to the pancreatic exocrine. Six male Sprague-Dawley rats (300 g) were randomly divided into two groups, a control group (C, n = 3) and a treatment group (T, 10.0μg/kg BW, n = 3). Blood samples were collected from rat caudal vein once time after one hour injection. The concentrations of plasma ghrelin, cholecystokinin (CCK) and alfa-amylase activity were evaluated by enzyme immunoassay (EIA) kit. Two-dimensional gel electrophoresis (2-DE) analysis was conducted to separate the proteins in pancreas tissue. Results showed that the i.p. infusion of ghrelin at doses of 10.0 μg/kg body weight (BW) increased the plasma ghrelin concentrations (p = 0.07) and elevated the plasma CCK level significantly (p < 0.05). Although there was no statistically significant, the α-amylase activity tended to increase. The proteomics analysis indicated that some pancreatic proteins with various functions were up- or down- regulated compared with control group. In conclusion, ghrelin may have role in the pancreatic exocrine, but the signaling pathway was still not clear. Therefore, much more functional studies focus on these found proteins are needed in the near future.

## Background

Ghrelin is a 28-amino-acid peptide isolated from the rat stomach in 1999 which is mainly produced by X/A-like cells in the oxyntic mucosa [1]. It was also found in other parts like hypothalamus, pituitary gland, lung, kidney [2–4], duodenum, ileum, colon and pancreas [5]. The ghrelin receptors (GHS-R) have been detected in many central and peripheral tissues and pancreatic α-cells, β-cells, exocrine cells. Ghrelin shows a number of actions at the gastrointestinal tract level. It is well known as a stronger activator of growth hormone (GH) through central nervous system (CNS) and modulation of food intake. And it also exhibits lots of other biological activities including energy expenditure, stimulation of lactotroph and corticotroph secretion, influence on sleep and behavior or modulation of heart rate and blood pressure ([6–9]; Assakawa et al. [10]).

Recent studies demonstrated that ghrelin could modulate exocrine secretions as well as pancreatic endocrine secretions. There were two opposite theories. First, Zhang and coworkers demonstrated that intravenous administration of ghrelin to the rats produced inhibition of enzyme secretion, and that this inhibitory effect of ghrelin on pancreatic exocrine secretion is indirect and may be exerted at the level of intrapancreatic neurons [11]. In contrast, other studies revealed that central as well as peripheral administration of ghrelin significantly increased pancreatic fluid and protein output, though the activation of vagal centers in the brainstem and stimulation of vagal efferent nerves [12, 13]. It is generally believed that, in the intestinal phase, stimulation of pancreatic enzyme secretion depends, in the main part, on the neuronal mechanism involved in the CCK release and activation of cholinergic vago-vagal enteropancreatic reflex. However, these physiological roles of ghrelin in the modulation of exocrine pancreatic functions are still unclear (Gherlaldoni et al. [14]).

Therefore, this study was conducted to determine the effects of exogenous ghrelin, given intraperitoneal (i.p.) on plasma CCK level and α-amylase output in the sprague-dawley rats. Meanwhile, the responses of pancreatic proteins to ghrelin were also analyzed using the 2-DE system.

## Methods

### Materials

Rat ghrelin peptide was obtained from Bachem (Bubendorf, Switzerland). Rat plasma ghrelin and CCK enzyme immunoassay kit were purchased from Phoenix Pharmaceuticals (Mountain View, CA, USA). The α-amylase activity analyzed using an EnzyChrom™ a-Amylase Assay Kit (ECAM-100).

### Animals

Sprague-Dawley rats (300 g) (Samtaco, Osan, Korea) were used for all experiments. Animals were housed at one animal per cage on a 12/12 h light cycle (lights on at 8 am) and given access to food and water ad libitum. Ghrelin (0.1, 1.0, 10.0 ug/kg, respectively) were injected intraperitoneally in rats. One hour after injection, blood samples (1 ml) were collected from rat caudal vein once time into heparined tube and immediately centrifuged (3,000 rpm/min, 15 min) to obtain the plasma. Aliquots of plasma were stored at -80°C till analyzed. Then rats were anaesthetized via intramuscular injection of zoletil (Vetbutal) at a dose of 15.0 mg/300 g (BW). And the rat pancreas tissues were collected. All experimental procedures were in accordance with the “Guidelines for the Care and Use of Experimental Animals of Pusan National University”.

### Determination of plasma ghrelin, CCK concentration and α-amylase activity

The plasma ghrelin and CCK concentration were separately determined by enzyme immunoassay kit (Phoenix Pharmaceuticals, Inc., Burlingame, CA, USA). The plasma α-amylase activity analyzed using an EnzyChrom™ a-Amylase Assay Kit (ECAM-100, BioAssay Systems, Hayward, CA, USA). All the operations were done followed the kit manual.

### Pancreas sample preparation and 2-DE analysis

Pancreatic tissues were collected and then pulverized into powder under liquid nitrogen and stored at -80°C until use. Tissues (0.5 g) were homogenized in 1ml lysis solution containing 7 M urea, 2 M thiourea, 4% chaps, 40 mM Tris, 65 mM DTT, 0.5% IPG buffer and 1X protease inhibitor (GE Healthcare, Piscataway, New Jersey, USA). This mixture was stirred every 5 min for 30 min and then centrifuged at 14,000 rpm for 30 min at 20°C. The supernatant was then stored in aliquots at -80°C until analysis.

2-DE was performed of pooling pancreatic tissue samples from three animals in each group. Briefly, protein samples were diluted into isoelectric focusing (IEF) buffer containing 6 M urea, 2 M thiourea, 1% 3-((3-Cholamidopropyl)dimethylammonium)-1-propanesulfonate (CHAPS), 0.002% bromophenol blue, 0.5% phamalyte (pH 3-10NL) and 65 mM dithiothreitol (DTT). Then 100 μg protein smaples of control or treatment was loaded on Immobiline DryStrip gels (pH 3-10NL, 18 cm, GE Healthcare) for rehydration for 12 h at 20°C. The IEF procedures were performed using an IEF electrophoresis unit (GE Healthcare) following the manufacturer’s protocols. The following voltage program was used after the 12 h rehydration: linear ramp from 500 to 1000 V over 2 h, then a constant voltage of 8000 V for 7 h to give a total of 56,000 V h. After focusing, gel strips were equilibrated in a solution containing 50 mM Tris/HCl (pH 8.8), 6 M urea, 2% SDS, 30% glycerol, 0.002% w/v bromophenol blue and DTT for 15 min, followed by incubation in the same solution but replacing DTT with 135 mM iodoacetamide (IAA) for another 15 min. After that the equilibrated strips were inserted into sodium dodecyl sulfate polyacrylamide gel electrophoresis (SDS-PAGE) gels (18 cm, 12%). SDS-PAGE was performed using an Ettan DALT 2-D gel system (GE Healthcare). Upon completion, gels were stained using a PluseOne Silver Staining Kit (GE Healthcare). The silverstained gels were scanned using an Umax scanner (PowerLook 2100XL, UMAX Technologies, Inc., Dallas, TX, USA). Scanned gel images were processed by Proteomweaver™ 2-D Analysis Software (Definiens AG, Munich, Germany).

### Statistical analysis

Data are presented as mean ± standard error of the mean (SEM). The group mean values were compared with an independent sample *t*-test (SPSS 14.0, Chicago, IL, USA). A *p*-value <0.05 was considered to be statistically significant.

## Results and discussion

The i.p. infusion of ghrelin at doses of 10.0 μg/kg body weight (BW) increased the plasma ghrelin concentrations (*p* = 0.07) and elevated the plasma CCK level significantly (*p* < 0.05). Although there was no statistically significant, the α-amylase activity tended to increase (Table 1). These data indicated that ghrelin may have role in pancreatic exocrine secretion through the stimulation of CCK release and activation of dorsal vagal nerve. CCK is one of the major gut hormones which released from duodenal mucosa I cells. It can stimulate pancreatic exocrine secretion via activation of CCK1 receptors and entero-pancreatic vago-vagal reflex [15–19]. Whether CCK plays a role in the exocrine secretion response to ghrelin is unknown.

**Table 1:** **Relationship between plasma G.I. hormones and α-amylase activity**

Items	Ghrelin (ng/ml)	CCK (ng/ml)	α-amylase (U/L)
Control^1^	0.202 ± 0.152^2^*	5.745 ± 2.428	3791.799 ± 208.308
Treatment^1^	3.223 ± 2.140	11.306 ± 3.937	4210.847 ± 262.825

^1^Control = vehicle/500 uLSaline, Treatment = Ghrelin (10.0 ug/kg)/500 uLSaline.

^2^Data are mean ± SEM and *means significantly different, p < 0.05 (Student’s paired *t*-test).

**Figure 1:**
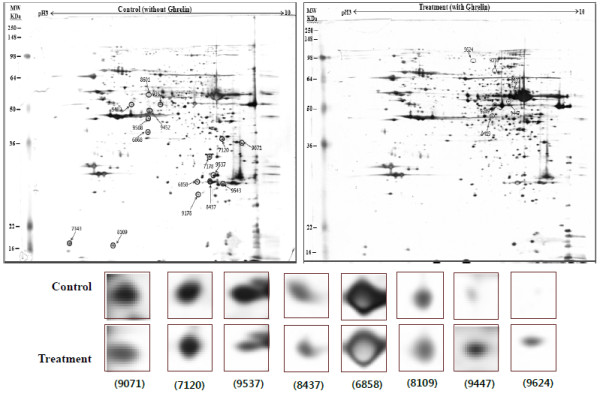
**Representative silver-stained 2-DE images of the normal rat pancreas (left) and treated rat pancreas by Ghrelin (right).**

The response of pancreatic proteins to ghrelin administration were analyzed by 2-DE in order to get a better understanding of the mechanisms involved in the ghrelin-exocrine secretion through CCK signal. Eight spots ranging from 6 to 200 kDa were detected differently expressed in the pancreatic protein map (Figure 1). One spot was up-regulated and six were down-regulated compared with control group. One spot was found only expressed in treatment group. These proteins were classified according to their functions (Table 2). Three down-regulated proteins eukaryotic translation initiation factor 5A-1 (eIF5A), peroxiredoxin IV (PRx IV) and ubiquinol-cytochrome c reductase (UQCR) were particular interested. eIF5A is involved in biosynthesis of pancreatic enzyme by increasing plasma CCK level [20]. And this data was different with ours. Judging by our reports, ghrelin may affect on pancreatic exocrine secretion via other factors as well as releasing CCK. Also, Yannick demonstrated that UQCR and PRx IV down-regulated biosynthesis of insulin related to diabetes [21]. Synthetically, when the effects of exogenous ghrelin, given intraperitoneal (i.p.) on pancreatic exocrine secretion, we have to consider other factor like growth hormone related to insulin synthesis as involving the CCK release and activation of cholinergic vago-vagal enteropancreatic reflex.

**Table 2:** **Identification of differentially altered protein spots in rat pancreas by ESI/Q-TOF MS**

Spot	Protein name	Peptide match	Protein score	Mass (bp)	Expression (treatment/control)
***Cell growth and proliferation***					
8109	Eukaryotic translation initiation factor 5A-1	295	98.23	16821.4	Down
***Gluconeogenesis***					
9071	Malate dehydrogenase, mitochondrial precursor	40	100.29	35660.8	Down
***De nove purine biosynthesis***					
9447	Similar to adenylosuccinate lyase	67	214.27	54817	Up
***Oxidative stress***					
6858	PRx IV	44	104.23	30988.1	Down
7120	Thiosulfate sulfurtransferase	392	154.28	33385.8	Down
9624	Sarcosine dehydrogenase, mitochondrial precursor	115	238.23	101578.9	Only Treatment
***Others***					
8437	Ubiquinol-cytochrome c reductase iron-sulfur subunit, mitochondrial precursor	55	100.32	29427.2	Down
9537	Carbonic anhydrase 3	297	148.26	29412.7	Down

## Conclusions

In conclusion, our result suggest a role of ghrelin on pancreatic exocrine, but the protein concerning with mechanism functional study was uncompleted. And some following functional study of proteins will be done in the near future.
